# Heat-killed VSL#3 Ameliorates Dextran Sulfate Sodium (DSS)-Induced Acute Experimental Colitis in Rats

**DOI:** 10.3390/ijms15010015

**Published:** 2013-12-19

**Authors:** Li-Xuan Sang, Bing Chang, Cong Dai, Nan Gao, Wei-Xin Liu, Min Jiang

**Affiliations:** 1 Department of Cadre Ward II, First Affiliated Hospital of China Medical University, Shenyang 110001, Liaoning, China; E-Mails: sanglixuan2008@163.com (L.-X.S.); liyuchun522@163.com (N.G.); 2 Department of Gastroenterology, First Affiliated Hospital of China Medical University, Shenyang 110001, Liaoning, China; E-Mails: cb000216@163.com (B.C.); congdai2006@sohu.com (C.D.); weixinliu66@163.com (W.-X.L.)

**Keywords:** probiotic, ulcerative colitis, inflammatory bowel disease, experimental colitis, *IL-6/STAT3*

## Abstract

To determine the effects of heat-killed VSL#3 (*B. breve*, *B. longum* and *B. infantis; L. plantarum*, *L. bulgaricus*, *L. casei* and *L. acidophilus; S. salivarius* subsp. *thermophilus*) therapy in the dextran sulfate sodium (DSS)-induced acute experimental colitis in rats. Acute experimental colitis was induced in rats by 5% DSS and freely drink for seven days. Beginning on Day 8, rats underwent gavage once daily for seven days with heat-killed probiotic VSL#3 (0.6 g/kg/day), colonic damage was evaluated histologically and biochemically seven days after gavage. Expression of inflammatory related mediators (*STAT3*, *P-STAT3*) and cytokines (*IL-6*, *IL-23*, *TGFβ*) in colonic tissue were detected. The results revealed that heat-killed and live VSL#3 have identical anti-inflammatory properties by the assessed DAI (disease activity index), colon length, histological tissue and MPO activity. Heat-killed and live VSL#3 results in reduced *IL-6*, *IL-23*, *TGFβ*, *STAT3* and *P-STAT3* expression in colonic tissue. Heat-killed and live VSL#3 have showed the similar anti-inflammatory activity by inhibiting *IL-6/STAT3* pathway in the DSS-induced acute experimental colitis in rats.

## 1. Introduction

Inflammatory bowel disease (IBD) is a set of chronic recurrent diseases, including ulcerative colitis (UC) and Crohn’s disease (CD). The exact pathogenic mechanism of IBD remains unclear but several possible mechanisms have been clarified. It has been shown that genetic factors, environmental factors, intestinal flora disturbance and immune dysregulation may involve in pathogenesis of IBD. More than four million people worldwide suffer from IBD. Currently, medical therapies for IBD include 5-aminosalicylic acid drugs, methotrexate, immunomodulators and biological therapies (mainly anti-tumor necrosis factor). The high cost, multiple adverse effects and limited effect prompt research and development of new treatment options.

Intestinal dysbacteriosis caused by bacterial invasion and changes in protective factors are involved in the pathogenesis of IBD. Changes in intestinal flora have been observed in dextran sulfate sodium (DSS)-induced acute experimental colitis, with the primary changes being increases in Bacteroides and Clostridia, suggesting that intestinal bacteria are involved in colitis in rats [1]. Moreover, transgenic or knockout methods result in immunodeficient animal models of IBD [2,3], however, in a sterile gut environment, IBD cannot develop in these models, nor can intestinal inflammation recur, suggesting that the composition of gut microbiota is necessary for the pathogenesis of IBD. However, many studies have shown that not all probiotic exert anti-inflammatory effects in rat model of colitis. Moreover, a feces enema was shown to ameliorate intestinal inflammation [4,5], and antibiotics have some beneficial effects in the treatment of IBD [6,7]. These relationships between IBD and intestinal bacteria suggest that normalizing intestinal microflora may be effective in the treatment of patients with IBD.

Probiotics are live microorganisms that provide beneficial effects to the host, and some studies demonstrated that probiotics ameliorate symptoms of IBD and relieve antibiotic-associate diarrhea. Moreover, probiotic VSL#3 was verified on successful induction and maintenance of the remission of ulcerative colitis. In addition to living bacteria, gamma-ray irradiated bacteria can play a useful role in experimental colitis. Similarly, extracted bacterial DNA and even the culture medium of bacteria can have the same effects, suggesting that the activity of probiotics may be mediated by their DNA [8]. However, the mechanisms by which heat-killed VSL#3 provoke beneficial effects are not yet fully understood. Moreover, VSL#3 treatment is not always effective for each patient of the intestinal inflammation, mainly because the efficacy of live probiotic may be influenced by the number probiotic that reach the intestinal lumen alive. In addition, the number of live probiotic may vary from one experiment to the next. Heat-killed probiotics can achieve stable effects because these are not required to colonize and maintain activity in the intestine. Therefore, we used heat-killed VSL#3 rather than live VSL#3 in our experiments.

To explore the effects of heat-killed VSL#3 on regular intestinal mucosal internal environment, we observed the level of the expression of *IL-6/STAT3* trans-signaling related cytokines in large intestine from rats with colitis induced by dextran sulfate sodium. *IL-6/STAT3* trans-signaling was shown to be activated in the intestinal mucosa of an IBD rat model, suggesting that signal transduction pathways play an important role in the pathogenesis of UC. Blocking this pathway may be useful in the treatment of UC. This study may provide a new strategy for the treatment of UC.

## 2. Results

### 2.1. Disease Activity Index, Colon Length, Histopathology and Score of Histopathological Lesions

DAI, colon length and histological score are shown in Table 1. In the DSS group, DAI decreased compared with those of mesalazine group, VSL#3 and heat-killed VSL#3 group. The DAIs of mesalazine and VSL#3 group was similar to heat-killed VSL#3 group (*p* > 0.05). In the live and heat-killed VSL#3 group, colon lengths were longer than those of the DSS group (*p* < 0.05) and were similar to mesalazine group (*p* > 0.05).

**Figure 1.**
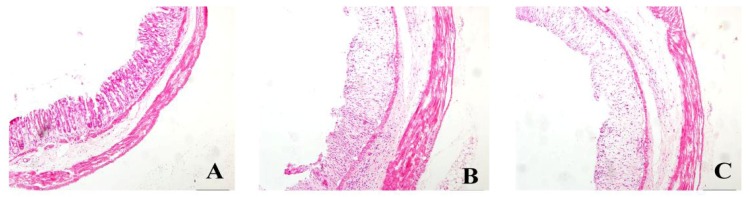

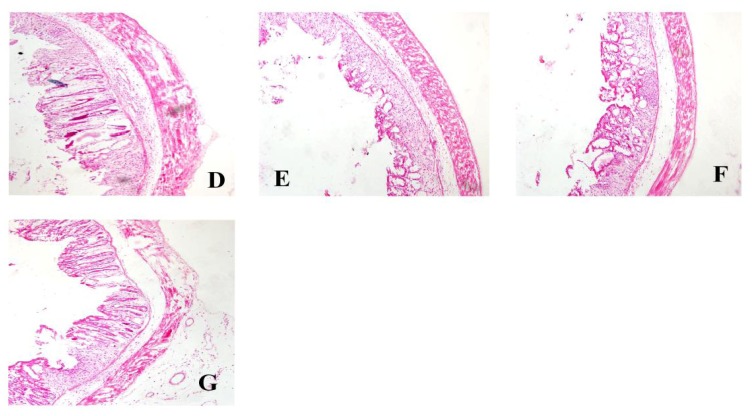
HE staining of rat colonic tissue samples (×10) from the **(A)** control group and **(B**–**G)** rats treated with DSS and (**C**) normal saline; (**D**) mesalazine; (**E**) VSL#3; (**F**) heat-killed VSL#3; and (**G**) mesalazine plus VSL#3.

**Figure 2.**
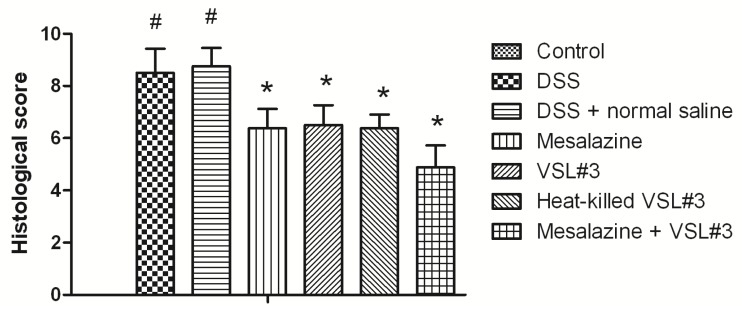
Histology injury score (^#^ *p* < 0.05 *versus* control group; * *p* < 0.05 *versus* DSS group).

Histopathologic changes were assessed by HE stain (Figure 1). Colonic membrane structure was intact in the control group. Extensive lesions were observed in DSS group, including mucosa destruction, crypt destruction and glands separation. mesalazine group, VSL#3 group, heat-killed VSL#3 group and VSL#3 + mesalazine group had significantly less histological damage compared with DSS group. Heat-killed VSL#3 group have reduced mucosal injury in experimental colitis. There was no significant difference between heat-killed VSL#3 group and mesalazine group (*p* > 0.05) (Figure 2).

### 2.2. MPO Activity

**Figure 3.**
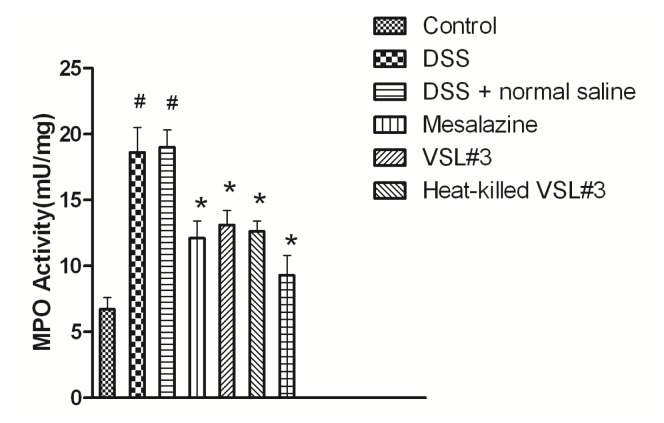
MPO activity of rats in each group (^#^ *p* < 0.05 *versus* control group; * *p* < 0.05 *versus* DSS group).

We assessed leukocyte infiltration in colonic tissue by determining the levels of MPO. The results of MPO are shown in Figure 3. MPO activity was significantly increased in the DSS group (18.6 ± 1.9 mU/mg) compared with the control group (6.7 ± 0.9 mU/mg) (*p* < 0.05). The MPO activity was significantly decreased by the mesalazine group (12.1 ± 1.3 mU/mg), VSL#3 group (13.1 ± 1.1 mU/mg), heat-killed VSL#3 group (12.6 ± 0.8 mU/mg) and VSL#3 + mesalazine group (9.3 ± 1.5 mU/mg) compared with DSS group (*p* < 0.05). Compared with mesalazine and VSL#3 group, MPO activity was similar to heat-killed VSL#3 group (*p* > 0.05).

### 2.3. Expression of IL-6, IL-23, TGF-β, and STAT3 mRNA in Colonic Tissue

**Figure 4.**
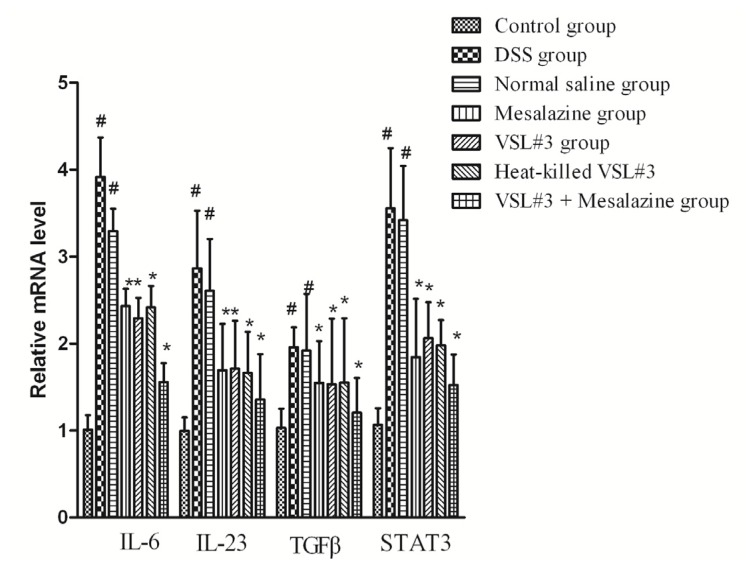
Relative expression of *IL-6*, *IL-23*, *TGF-β*, and *STAT3* mRNA in groups of rats. Expression was assessed by real-time RT-PCR. Groups of rats are defined in the text (^#^ *p* < 0.05 *versus* control group; * *p* < 0.05 *versus* DSS group).

The mRNA expression levels of *IL-6*, *IL-23*, *TGF-β*, and *STAT3* were examined using real-time PCR. The mRNA levels of these genes in the DSS group were significantly higher than in the control group (*p* < 0.05). Heat-killed VSL#3 group significantly reduced the mRNA levels in DSS-induced colons compared with the DSS group (*p* < 0.05). The mRNA levels of these genes in the heat-killed VSL#3 group were no significant differences in the mesalazine and VSL#3 group (*p* > 0.05) (Figure 4).

### 2.4. Western-Blot Analysis

**Figure 5.**
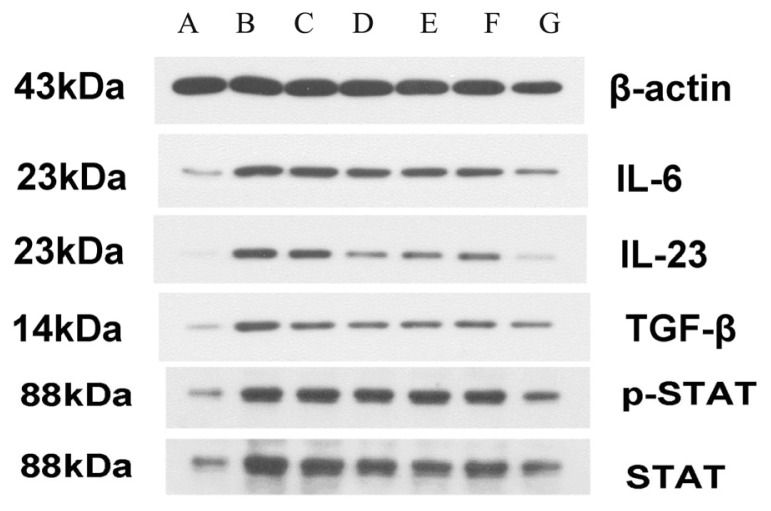
Western blots of *IL-6*, *IL-23*, *TGF-β*, *STAT3*, and *P-STAT3* in each group (Lane **A** control group; Lane **B** DSS group; Lane **C** placebo group; Lane **D** Mesalazine group; Lane **E** VSL#3 group; Lane **F** Heat-killed VSL#3 group; Lane **G** VSL#3 + Mesalazine group).

**Figure 6.**
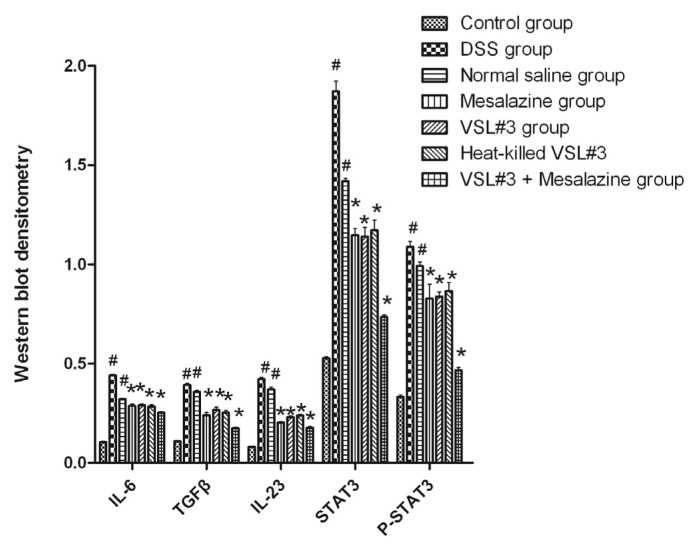
Western blot densitometry of the proteins *IL-6*, *IL-23*, *TGF-β*, *STAT3*, and *P-STAT3* in colon tissue samples from groups of rats (^#^ *p* < 0.05 *versus* control group; * *p* < 0.05 *versus* DSS group.

We examined the effect of probiotic VSL#3 and heat-killed VSL#3 on the protein expression of *IL-6*, *IL-23*, *TGF-β*, *STAT3* and *P-STAT3* proteins in the colons of rats of each group by Western-blot. In contrast to control group, the protein expression of *IL-6*, *IL-23*, *TGF-β*, *STAT3* and *P-STAT3* were significant increased in DSS group (*p* < 0.05). In contrast to DSS group, the protein of expression of heat-killed VSL#3 group were significant decreased (*p* < 0.05) and were similar to mesalazine and VSL#3 group (*p* > 0.05) (Figures 5 and 6).

## 3. Discussion

Many chemical compositions have been used to induce experimental colitis, such as TNBS [9], acetic acid [10], OXZ [11] and DSS [12]. DSS-induced colitis is widely used to evaluate the effects of drugs because of its similarity to UC [13], and low mortality [14]. Therefore, DSS-induced colitis is adopted in our study.

In this study, we observed the effects of heat-killed VSL#3 on acute experimental colitis induced by DSS. Our results showed that heat-killed VSL#3 could reduce the colon injury, elevated shorter colon length, decrease DAI and MPO activity, as well as expression of *IL-6*, *TGF-β*, *STAT3* and *P-STAT3*. Live VSL#3 are able to maintain the intestinal homeostasis as well as to ameliorate intestinal disorders. However, live VSL#3 must be colonize the intestinal lumen and maintain their activities when exerting its physiological functions, while heat-killed VSL#3 requires no colonization in the intestinal lumen. Therefore, heat-killed VSL#3 are hoped to be effective even though the intestinal condition is not conducive to colonizing and maintaining their activities.

Madsen *et al.* [15] have reported that oral administration of VSL#3 was effective as primary therapy in *IL-10* gene-deficient mice, and had a direct effect on epithelial barrier function. Rachmilewitz *et al.* [8] have reported that the administration of heat-killed VSL#3 (100 °C for 30 min) had no effect on the severity of DSS induced colitis and this results are in contrast with ours (121 °C for 20 min). A heat-killed may cause structural or chemical change in the composition of immunomodulatory substance in VSL#3 which may lead to changes in the composition and function of cytokines. The different temperature and period may result in different results. Furthermore, strain of animal, dose of VSL#3, severity of colitis may also lead to different results.

Oxidative stress is believed to play an important role in the pathogenesis of colitis-related intestinal tissue injury [16]; MPO is a marker of oxidative stress produced mainly by polymorphonuclear leucocytes and is associated with the severity of colitis. The assessment of MPO activity is well established for evaluating intestinal inflammation [17,18]. Therefore, we assessed leukocyte infiltration in colonic tissue by detecting the levels of MPO. In our results, MPO activity was significantly decreased in heat-killed VSL#3 group compared with the DSS group. Therefore, heat-killed VSL#3 suppressed inflammation induced by DSS by suppressing the MPO-mediated activation of inflammatory cells.

The hyper-activation of immune cells involved in the pathogenesis of ulcerative colitis, known to produce high levels of pro-inflammatory cytokines such as *IL-6. IL-6* is a multifunctional cytokines that plays an important role in the initiation and continuation of mucosal inflammation [19,20].

The study suggested that an increase serum-6 during active IBD [21]. Moreover, RT-PCR analysis showed that *IL-6* mRNA levels are increased in active IBD patients [22]. In our study, *IL-6* levels were associated with the severity of inflammation. *IL-6* levels significantly increased in the DSS group. Compared with the DSS group, *IL-6* levels significantly decreased in heat-killed VSL#3 group. Anti-*IL-6* was proposed as anti-inflammatory agent in IBD has been reported [23]. The results of our study suggest the heat-killed VSL#3 decreased the expression of pro-inflammatory cytokines *IL-6*.

The *IL-23* pathway has demonstrated significant advances in the UC and plays a key role in mediating intestinal inflammation, host defense and autoimmunity. *IL-23* comes from a variety of cells during intestinal inflammation, including monocyte-derived cells [24]. Moreover, transgenic expression of *IL-23* leads to severe intestinal inflammation [25]. *IL-23* blockade or deficiency protects from intestinal inflammatory disease [26,27], including human IBD [28]. The association of *IL-23* receptor mutations with CD in a genome-wide association study has suggested that *IL-23* is a key role in human IBD [29]. In our study, *IL-23* levels were associated with the severity of inflammation. *IL-23* levels significantly decreased in heat-killed VSL#3 group compared with the DSS group. Anti-*IL-23* was proposed as anti-inflammatory agent in IBD has been reported [23]. The current study has shown that heat-killed VSL#3 decrease the expression of *IL-23*.

*TGFβ* is a pleiotropic cytokine with important functions for the maintenance of immune balance. The study showed that *TGFβ* is very important to control T Cell periphery [30]. Recent investigations have demonstrated that the role of *TGFβ* in suppressing of T cell mediated autoimmune inflammation [31,32]. However, *TGFβ* and *IL-6* can drive the initial differentiation of *IL-17A* and lead to colon inflammation [33]. Therefore, *TGFβ* can play both anti- and pro-inflammatory roles in the immune system. In our study, *TGFβ* levels were associated with the severity of colon inflammation. *TGFβ* levels significantly increased in the DSS group. *TGFβ* levels significantly decreased in VSL#3 group, heat-killed VSL#3 and VSL#3 + mesalazine group compared with the DSS group. *TGFβ* was proposed as pro-inflammatory agent in our experiment and the exact mechanism of its remains unclear.

Recent studied have revealed dextran sulfate sodium-induced colitis was mild in mice lacking *STAT3* in macrophages and gut epithelial cells [34]. Moreover, the role of *STAT3* in IBD has been documented by human IBD studies [35] and animal models of colitis [36]. Mudter *et al.* showed that an increase amount of *STAT3* protein appeared in IBD compared to control cell [37]. Furthermore, higher expression of phosphorylated *STAT3* was associated with the disease activity in animal models of colitis as well as in IBD patients [36].

The results of our study indicated *STAT3* and phosphorylated *STAT3* levels were associated with the severity of inflammation. *STAT3* and phosphorylated *STAT3* levels significantly increased in the DSS group. By contrast, *STAT3* and phosphorylated *STAT3* levels significantly decreased in heat-killed VSL#3 group. The results of our study suggest the therapeutic potential of heat-killed VSL#3 with increase *STAT3* and phosphorylated *STAT3* levels. The therapeutic effects of heat-killed VSL#3 on colitis may due to its effects on *IL-6/STAT3* pathway. Furthermore, some possible mechanisms of intestinal protection by probiotics have been proposed, including the up-regulation of tight junction proteins ZO-1 expression [38], activated the p38MAPK pathway, regulated the production of proinammatory cytokines, improved the barrier function of the intestinal epithelia in the presence of oxidant stress by heat shock protein [39], increased production of IgA [40] and inhibited the growth of pathogenic bacteria as antibiotic [41].

Our study raises the following questions: (i) is heat-killed VSL#3 effective on other drug induced colitis, such as TNBS and OXZ-induced colitis? (ii) is heat-killed VSL#3 effective on other intestinal diseases, such as irritable bowel syndrome? (iii) what are the specific mechanisms of heat-killed VSL#3 when exerting its physiological functions? and (iv) are other probiotic species also efficacious? Further studies are needed to elaborate these questions.

## 4. Materials and Methods

### 4.1. Animals

Male SD rats (Eight-week-old), weighing 180 ± 10 g, were purchased from Experimental Animal Center of China Medical University (Shenyang, China), rats were housed in the SPF laboratory. The rats were allowed to adapt to their environment for 1 week, with temperature maintained at 21 ± 1 °C, with 12 h of light (08:00–20:00) and 12h of dark (20:00–08:00) per day and 60%–80% relative humidity. All animal experiments confirmed to the animal protection laws in China and were approved by the China Medical University Animals Committee (Shenyang, China).

### 4.2. Experimental Protocol

The VSL#3 mixture combines 8 strains of lactic acid producing bacteria, including three strains of *Bifidobacterium* (*B. breve*, *B. longum* and *B. infantis*); four strains of *Lactobacillus* (*L. plantarum*, *L. bulgaricus*, *L. casei* and *L. acidophilus*) and one strain of *Streptococcus* (*S. salivarius* subsp. *thermophilus*). Each 2.5 g VSL#3 sachet contained 450 billion freeze-dried bacteria. Heat-killed VSL#3 was prepared by incubating VSL#3 at 121 °C for 20 min.

### 4.3. Induction of Acute DSS Colitis

Dextran sodium sulfate (DSS, Wako Pure Chemical Industries, Ltd, Osaka, Japan; molecular weight 5000 d), was dissolved in drinking water to a concentration of 5% DSS for 7 days and shifted to normal drinking water.

### 4.4. Experimental Protocol

Fifty-six SD rats were randomized into 7 groups (*n* = 8). Eight rats without pretreatment served as controls. In the DSS group, acute experimental colitis was induced as described above. In the placebo group, 200 μL normal saline was applied via gastric tube. In the live and heat-killed VSL#3 group, acute experimental colitis was induced as described above, eight rats were administrated with VSL#3 or heat-killed VSL#3 (0.6 g/kg/day) via gastric tube after induction of colitis. The mesalazine group was treated with 0.4 g/kg/day of mesalazine via gastric tube after induction of colitis. The VSL#3 + mesalazine group was treated with VSL#3 (0.6 g/kg/day) and mesalazine (0.4 g/kg/day) in the same manner. The dose of drug was according to body weight of rats at that time.

### 4.5. Sample Collection

All rats were injected with hydrated chloral and sacrificed after anesthesia. Their colons were washed with precooled saline, and a 0.5 cm portion of the terminal ileum was clipped, soaked in 4% formaldehyde, embedded in paraffin sections, stained with hematoxylin and eosin, and examined histologically. The remainder of each specimen was stored at −80 °C.

### 4.6. Assessment of Colitis

Colitis was evaluated by disease activity index (Table 2), with a modification by fecal property, fecal occult blood and body weight loss (the integral of each project sum up to a total DAI score). The length of the colon were measured and histological injury score of each colon was evaluated independently by two experienced pathologists (Table 3), with any differences settled by consultation with a third pathologist (the integral of each project sum up to a total histological injury score).

### 4.7. RNA Isolation and Quantitative Real-Time Polymerase Chain Reaction (PCR)

Total RNA was isolated from colonic tissue samples and subjected to the synthesis of cDNA. The individual cDNA species were amplified and PCR was performed by a real-time PCR system (ABI 7500, Foster, CA, USA). Primer sequences included those for mouse *β-actin* (5′-ACGTTGACAT CCGTAAAGAC-3′ and 5′-GAAGGTGGACAGTGAGGC-3′), *IL-6* (5′-CGCAAAGCCAGAGT CATTC-3′ and 5′-TGGTCTTGGTCCTTAGCC-3′), *IL-23* (5′-ACCTGCTGGACTCGGACAT-3′ and 5′-GGCGAGGCATCTGTTGAT-3′), *STAT3* (5′-CAAGGGCTTCTCGTTCTG-3′ and 5′-GGGCTT TGTGCTTAGGAT-3′), and *TGFβ* (5′-CAATTCCTGGCGTTACCT-3′ and 5′-AAAGCCCTGTA TTCCGTC-3′). The amplification protocol consisted of an initial denaturation at 95 °C for 5 min, followed by 30 to 32 cycles of denaturation at 95 °C for 10 s, annealing at 56 °C for 20 s, and extension at 68 °C for 40 s, followed by a final extension at 68 °C for 5 min.

### 4.8. Western Blotting Analysis

In order to determine the levels of *IL-6*, *IL-23*, *TGFβ*, *STAT3* and *P-STAT3*. Colorectal tissue were homogenized at 4 °C and supernatants after centrifugation at 14,000 *g* for 15 min, protein were subjected to SDS-PAGE before blotting onto a polyvinylidene difluoride membrane. The membrane was blocked with in a block buffer (5% skim milk) for 2 h at room temperature.

### 4.9. Statistical Analysis

All statistical analyses were performed using SPSS 16.0 software (SPSS Inc, Chicago, IL, USA). Data were expressed as mean ± standard deviation and compared by one-way ANOVA. A *p*-value < 0.05 was considered statistically significant.

## 5. Conclusions

In summary, our study showed that heat-killed VSL#3 ameliorate DSS-induced colitis in SD rats through its immunomodulatory activities. Heat-killed VSL#3 improved DAI, colon length and histological tissue injury in the DSS-induced acute experimental colitis. Moreover, heat-killed VSL#3 improved inflammation by suppressing MPO activation and decreasing *IL-6* concentrations. Therefore, further studies are needed to elaborate the mechanisms of suppression of the *IL-6/STAT3* pathway. Heat-killed VSL#3 may be a safer method than using live VSL#3 in some special conditions such as immunosuppressed individuals in future.

## Figures and Tables

**Table 1.** The effect of each group on DAI, colon length and histopathological score in DSS-induced acute experimental colitis.

Group	DAI	Colon length	Histopathological score
Control	0.12 ± 0.5 bc	19.09 ± 0.41 bc	0 bc
DSS	7.75 ± 0.75 ac	15.62 ± 0.39 ac	8.50 ± 0.93 ac
DSS + normal saline	7.12 ± 0.64 ac	15.59 ± 0.41 ac	8.75 ± 0.71 ac
Mesalazine	6.00 ± 0.93 ab	17.38 ± 0.47 ab	6.38 ± 0.74 ab
VSL#3	6.25 ± 0.71 ab	17.05 ± 0.33 ab	6.50 ± 0.76 ab
Heat-killed VSL#3	6.12 ± 0.84 ab	16.95 ± 0.47 ab	6.38 ± 0.52 ab
Mesalazine + VSL#3	5.00 ± 0.76 abc	18.05 ± 0.58 abc	4.88 ± 0.84 abc

a *p <* 0.05 compared with the control group;

b *p <* 0.05 compared with the model group;

c *p <* 0.05 compared with the mesalazine group.

**Table 2.** DAI score chart.

Fecal Property	Fecal Occult Blood	Body Weight Decrease (%)	Integral
Normal	Normal	0	0
		1–5	1
Relaxed	Positive Fecal Occult Blood	>5–10	2
		>10–15	3
Loose stools	Naked Eye Fecal Occult Blood	>15	4

Normal stool, shaped stool; relaxed stool, pasty, unformed stools not attached to the anus; loose stools, unshaped stools attached to the anus.

**Table 3.** Histology injury score chart.

Integral	0	1	2	3	4
Inflammation	None	Mild	Moderate	Severe	-
Mucosal damage	None	Mucous layer	Submucosa	Muscularis and serosa	-
Crypt damage	None	1/3	2/3	100%	100% with epithelium loss
Pathological change range	None	0%–25%	26%–50%	51%–75%	76%–100%
